# Real-world therapeutic trajectories in pustular psoriasis: Outcomes and relapse patterns in a low-resource health system

**DOI:** 10.1016/j.jdin.2026.02.008

**Published:** 2026-03-06

**Authors:** Diana Espitia-Hernandez, Elkin Peñaranda-Contreras, Carolina Cortes-Correa

**Affiliations:** a Fundación Universitaria Sanitas, Bogotá, Colombia; b Dermatology Department, Hospital Universitario de la Samaritana, Bogotá, Colombia

**Keywords:** biological therapy, epidemiology, health services accessibility, pustular psoriasis, recurrence, treatment outcome

*To the Editor:* Pustular psoriasis comprises a heterogeneous group of inflammatory dermatoses associated with significant morbidity, recurrent flares, and complex therapeutic decision-making.^1^ Although biologic therapies have improved outcomes in severe forms, most real-world evidence originates from high-income settings with stable access to advanced treatments.^2^^,^^3^ Data describing long-term treatment trajectories in resource-limited health systems remain scarce. We sought to characterize treatment patterns, therapeutic escalation, and relapse behavior among patients with pustular psoriasis managed in a national referral center within a middle-income country.

We conducted a retrospective observational study including patients diagnosed with any clinical variant of pustular psoriasis between January 2014 and December 2025. Medical records were reviewed for demographic characteristics, clinical subtype, time to specialist evaluation, histopathologic confirmation, systemic and biologic treatments across therapeutic lines, and relapse patterns. All data were anonymized, and follow-up duration varied according to available clinical encounters.

Fifty patients were included, the majority of whom presented with generalized pustular psoriasis, although localized and mixed variants were also observed (Table I). Diagnostic delay was common, with frequent initial misdiagnoses in emergency or primary care settings. Relapses occurred in 33 patients (66%), with a mean of 1.72 relapses per patient and a mean relapse duration of 4.24 months (Table I). Emotional stress was the most frequently identified trigger.

**Table I:** Demographic, clinical, and relapse characteristics of patients with pustular psoriasis (*N* = 50)

Variable	Value
Female sex, *n* (%)	32 (64%)
Age, y, mean ± SD	31.26 ± 15.43
Median age, y	25.5
Clinical subtype, *n* (%)	
Generalized pustular psoriasis	41 (82%)
Other pustular variants∗	9 (18%)
Prior plaque psoriasis, *n* (%)	18 (36%)
Initial emergency department consultation, *n* (%)	28 (56%)
Initial misdiagnosis, *n* (%)	
Impetigo	9 (18%)
Adverse drug reaction	7 (14%)
Folliculitis	5 (10%)
Skin biopsy performed, *n* (%)	49 (98%)
Relapse characteristics	
Patients with ≥ 1 relapse, *n* (%)	33 (66%)
Mean number of relapses ± SD	1.72 ± 0.97
Median number of relapses	2
Range of relapses	0-4
Mean time to relapse, months ± SD	4.24 ± 2.00
Most frequently identified trigger, *n* (%)	Emotional stress (15%)

*SD*, Standard deviation.

∗ Includes localized and mixed pustular psoriasis variants.

Conventional systemic therapies predominated early management (Table II). Cyclosporine was the most frequently used first-line agent (42%), followed by systemic corticosteroids (16%) and methotrexate (4%). However, these treatments demonstrated limited durability, as evidenced by frequent dose escalation, treatment reinitiation, or substitution in subsequent therapeutic lines. Methotrexate emerged as the most common second-line therapy (30.6%), reflecting both clinical practice patterns and restricted access to biologic agents. Despite its widespread use, methotrexate was often discontinued because of incomplete disease control or delayed therapeutic response.

**Table II:** Distribution of systemic and biologic treatments across therapeutic lines in pustular psoriasis (*N* = 50)

Treatment	First line, *n* (%)	Second line, *n* (%)	Third line, *n* (%)
Cyclosporine	21 (42.0)	6 (16.7)∗	2 (10.0)
Cyclosporine + acitretin	7 (14.0)	—	—
Cyclosporine + methotrexate	3 (6.0)	—	—
Acitretin (monotherapy)	1 (2.0)	3 (8.3)	1 (5.0)
Acitretin (dose escalation)	—	1 (2.8)	1 (5.0)
Prednisone	7 (14.0)	—	—
Prednisone + acitretin	1 (2.0)	—	—
Methotrexate	2 (4.0)	11 (30.6)	—
Methylprednisolone + methotrexate	1 (2.0)	—	—
Secukinumab	—	3 (8.3)	7 (35.0)
Ixekizumab	—	4 (11.1)	2 (10.0)
Guselkumab	—	2 (5.6)	2 (10.0)
Ustekinumab	—	1 (2.8)	2 (10.0)
Infliximab	—	1 (2.8)	—
Tofacitinib	—	1 (2.8)	—
Cyclosporine (restart or dose increase)	—	6 (16.7)	—
Topical therapy added	—	1 (2.8)	2 (10.0)
Calcipotriol/betamethasone	3 (6.0)	1 (2.8)	—
Clobetasol	4 (8.0)	—	—
Loss to follow-up	—	—	1 (5.0)

∗ Includes cyclosporine restart or dose escalation.

Biologic therapies were primarily introduced in later treatment lines and demonstrated greater treatment persistence. Interleukin (IL) 17 inhibitors, particularly secukinumab and ixekizumab, were the most frequently sustained biologic agents, followed by IL-23 and IL-12/IL-23 inhibitors (Table II). Once initiated, biologic therapies were less frequently replaced compared with conventional systemic agents. Nevertheless, treatment interruption remained common, largely due to access limitations rather than lack of efficacy.

These findings illustrate the clinical complexity of managing pustular psoriasis in resource-limited settings, where therapeutic decisions are strongly influenced by structural barriers to biologic access. Although biologics demonstrated superior durability, inconsistent availability necessitated continued reliance on conventional systemic agents, particularly methotrexate, as essential alternatives.^4^ Earlier recognition of pustular psoriasis and policies that improve sustained access to biologic therapies are critical to reducing relapse burden and improving long-term outcomes.^5^

This study has limitations inherent to its retrospective, single-center design. Follow-up duration varied across patients, and treatment decisions were influenced by restricted access to biologic therapies, which may limit generalizability to higher-resource settings. Nevertheless, the study provides valuable real-world insight into long-term management of pustular psoriasis in a low-resource health system.

## Conflicts of interest

None disclosed.
